# Expression and processing of the Hepatitis E virus ORF1 nonstructural polyprotein

**DOI:** 10.1186/1743-422X-3-38

**Published:** 2006-05-26

**Authors:** Deepak Sehgal, Saijo Thomas, Mahua Chakraborty, Shahid Jameel

**Affiliations:** 1 Virology Group, International Center for Genetic Engineering and Biotechnology, Aruna Asaf Ali Marg, New Delhi 110 067, India

## Abstract

**Background:**

The ORF1 of hepatitis E virus (HEV) encodes a nonstructural polyprotein of ~186 kDa that has putative domains for four enzymes: a methyltransferase, a papain-like cysteine protease, a RNA helicase and a RNA dependent RNA polymerase. In the absence of a culture system for HEV, the ORF1 expressed using bacterial and mammalian expression systems has shown an ~186 kDa protein, but no processing of the polyprotein has been observed. Based on these observations, it was proposed that the ORF1 polyprotein does not undergo processing into functional units. We have studied ORF1 polyprotein expression and processing through a baculovirus expression vector system because of the high level expression and post-translational modification abilities of this system.

**Results:**

The baculovirus expressed ORF1 polyprotein was processed into smaller fragments that could be detected using antibodies directed against tags engineered at both ends. Processing of this ~192 kDa tagged ORF1 polyprotein and accumulation of lower molecular weight species took place in a time-dependent manner. This processing was inhibited by E-64d, a cell-permeable cysteine protease inhibitor. MALDI-TOF analysis of a 35 kDa processed fragment revealed 9 peptide sequences that matched the HEV methyltransferase (MeT), the first putative domain of the ORF1 polyprotein. Antibodies to the MeT region also revealed an ORF1 processing pattern identical to that observed for the N-terminal tag.

**Conclusion:**

When expressed through baculovirus, the ORF1 polyprotein of HEV was processed into smaller proteins that correlated with their proposed functional domains. Though the involvement of non-cysteine protease(s) could not be be ruled out, this processing mainly depended upon a cysteine protease.

## Background

Hepatitis E virus (HEV) is the etiological agent for hepatitis E. It has been the cause of large epidemics as well as many sporadic cases of acute viral hepatitis in much of the developing world [1-5]. The viral genome is a single-stranded 7.2-kb polyadenylated RNA of positive sense containing three open reading frames (ORFs) [6,7]. Of these, ORF2 encodes an 88-kDa glycoprotein that is the major viral capsid protein [8,9]; ORF3 encodes a phosphoprotein [10], which is involved in cell signaling through MAP kinase pathway [11].

**Figure 1:**
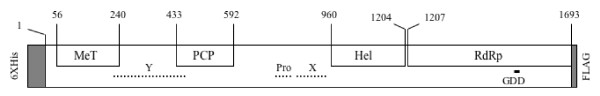
**The HEV ORF1 polyprotein**. A schematic illustration of the HEV ORF1 nonstructural polyprotein is shown, with the engineered N- and C-terminal tags. The predicted methyltransferase (MeT), papain-like cysteine protease (PCP), helicase (Hel) and RNA dependent RNA polymerase (RdRp) domains are shown, as is the GDD sequence that forms the RdRp active site. The numbers on top represent amino acids of the predicted domains numbered according to the ORF1 polyprotein sequence [12]. The Y, proline-rich (Pro) and X regions with no predicted function are also shown. The tags engineered at the two ends include the N-terminal 6XHis tag of 45 amino acids (from vector pBBHis-2b) and a FLAG epitope of 12 amino acids as described in Materials and Methods. The entire recombinant ORF1 polyprotein engineered here is expected to be 1760 amino acids long, with a predicted mass of 191,806 Da.

The third ORF, called ORF1 is 5109 bp long and encodes the viral nonstructural polyprotein with a proposed molecular mass of ~186 kDa. Based on protein sequence homology, the ORF1 polyprotein is proposed to contain four putative domains indicative of methyltransferase (MeT), papain-like cysteine protease (PCP), RNA Helicase (Hel), and RNA dependent RNA polymerase (RdRp) (Fig. 1) [12]. Of these, the MeT and RdRp enzymatic activities have been demonstrated [13,14] while activities of the Hel and PCP have so far not been elucidated. Attempts have also been made to study ORF1 processing using different expression systems. In one study, the ~186 kDa ORF1 polyprotein was expressed through recombinant vaccinia virus infection of mammalian cells, but no processed products were initially observed [15]. Following extended incubation for 24–36 hours, two processed bands of ~107 and ~78 kDa were observed. Mutagenesis of the proposed cysteine protease domain of ORF1 suggested that the HEV protease had no role in ORF1 polyprotein processing. The cleavage of the ~186 kDa protein was attributed either to a vaccinia-virus encoded protease or a cellular protease.

In another study, ORF1 processing was addressed through *in vitro *transcription and translation, and expression in either *E. coli *or human cells [16]. Prokaryotic expression resulted in a ~212 kDa glutathione-S-transferase fusion protein that exhibited strong reactivity with the antibodies raised against the putative domains of ORF1. Since no other smaller products were observed, ORF1 processing did not seem to occur in the prokaryotic system. When the expression of ORF1 was studied by carrying out *in vitro *coupled transcription and translation, a polyprotein of ~186 kDa could again be immunoprecipitated with antibodies against the various putative domains of ORF1, but no smaller fragments were observed. The expression in transiently transfected HepG2 cells also resulted in a ~186 kDa protein, but no other smaller sized fragments were seen [16]. Transfection of an *in vitro *generated infectious full-length HEV RNA into HepG2 cells has also been used to assess ORF1 expression and processing [17]. This resulted in the formation of processed forms of the ORF1 polyprotein that could be immunoprecipitated with various domain-specific antibodies [17].

Though ORF1 processing was reported in at least one study in the context of genomic RNA, it is not clear why this was not observed in other studies. This could be due to improper folding of the GST-ORF1 fusion protein expressed in the prokaryotic system, and low yields of the protein expressed in coupled *in vitro *transcription-translation or mammalian expression systems. To address this, we expressed a recombinant ORF1 polyprotein tagged at its N- and C-termini in insect cells using a baculovirus expression system, and detected the processed fragments using antibodies specific for the N-terminal hexa-histidine and C-terminal FLAG epitopes. Using this strategy, we show here that the ORF1 polyprotein is processed in insect cells and that this involves both cysteine and non-cysteine proteases. The processing of ORF1 was also confirmed by mass spectrometric analysis of one of the processed fragments and by western blotting with antibodies to the methyltransferase domain.

## Results

### Construction of the recombinant baculovirus

The HEV-ORF1 was PCR amplified (data not shown) using the HEV full-length cDNA as a template, so that when expressed, the protein had an N-terminal hexa-histidine tag and a C-terminal FLAG tag (Fig. 1). The amplified gene was cloned in TOPO-TA vector, *in vitro *transcribed and translated to generate a polyprotein of ~192 kDa (data not shown). After confirming proper expression of the amplified fragment, it was cloned in the baculovirus transfer vector pBlueBacHis-2b (Invitrogen). Co-transfection of the recombinant plasmid and pBlueBac DNA (Invitrogen), followed by selection and plaque purification, resulted in generation of the recombinant virus, called vORF1. For subsequent infection, this was amplified to a titer of 10^8 ^pfu/ml in *Sf21 *cells.

### ORF1 expression and processing

**Figure 2:**
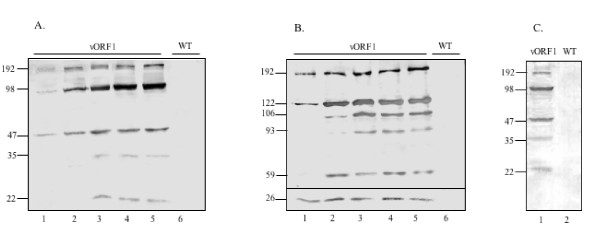
**Expression and processing of the ORF1 polyprotein**. *T. ni *cells infected with the vORF1 recombinant virus were harvested at various times post-infection and the lysates subjected to SDS-PAGE and western blotting with anti-His (A) or anti-FLAG (B) antibodies. Lanes 1 to 5 show results at 24, 36, 48, 60 and 72 hr post-infection; lane 6 shows the result for wild type AcMNPV infection after 48 hr. Panel C shows the 48 hr lysate probed with anti-MeT antibodies. In (B) the upper and lower panels show results from 7.5% and 12% SDS-polyacrylamide gels. The estimated fragment sizes are shown based on molecular size markers run on each gel (not shown).

To study the time course of recombinant ORF1 polyprotein expression, vORF1 was used to infect *T. ni *cells. The infected cells were harvested at various times post-infection and the lysates subjected to SDS-PAGE followed by western blotting using anti-His or anti-FLAG antibodies (Fig. 2). Expression of the ORF1 polyprotein was seen as early as 24 hr post-infection (hpi) (Fig. 2A, lane1), the time at which the polyhedrin promoter is activated. At this time, besides the ~192 kDa fragment, other fragments with sizes of ~98 and 47 kDa were also observed with anti-His antibodies. Around 48 hpi, two additional bands of ~35 and 22 kDa were seen and all of these fragments were found to increase with time till 72 hpi (Fig. 2A). When expression was analyzed using anti-FLAG antibodies, besides the ~192 kDa polyprotein, smaller fragments of ~122, 106, 93, 59 and 26 kDa were also observed in a temporal manner (Fig. 2B). As a negative control, no staining was observed with either antibody in wild type AcMNPV (wt) infected *T. ni *cells (Fig. 2A and 2B, lane 6). The expression of the ~192 kDa fragments and accumulation of smaller fragments as a function of time was indicative of processing of the ORF1 polyprotein. The processing was further confirmed with antibodies against the MeT domain, the most N-terminal predicted domain in the polyprotein. The pattern of processing observed with anti-MeT antibodies (Fig. 2C) was identical to that obtained using anti-His antibodies (Fig. 2A).

### Effect of cysteine protease inhibition on ORF1 processing

**Figure 3:**
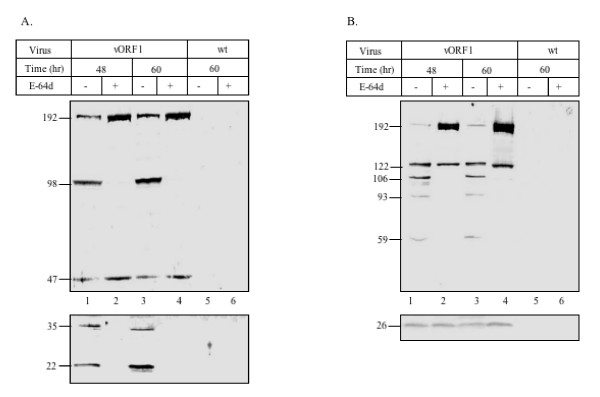
**Effect of E-64d on ORF1 polyprotein processing**. T. ni cells were infected with vORF1 for 12 hr at which time fresh medium containing 200 μM E-64d was added to the cells; an equal volume of DMSO served as the control. At 48 and 60 hr following E-64d addition, cells were harvested and the lysates analyzed by western blotting with either anti-His (A) or anti-FLAG (B) antibodies (lanes 1–4). Lysates from wild type AcMNPV infected cells were similarly analyzed at 48 hr after E-64d addition (lanes 5–6). In both parts, the upper and lower panels show results obtained following separation of the proteins on 7.5% and 12% SDS-polyacrylamide gels. The estimated fragment sizes are shown based on molecular size markers run on each gel (not shown).

**Figure 4:**
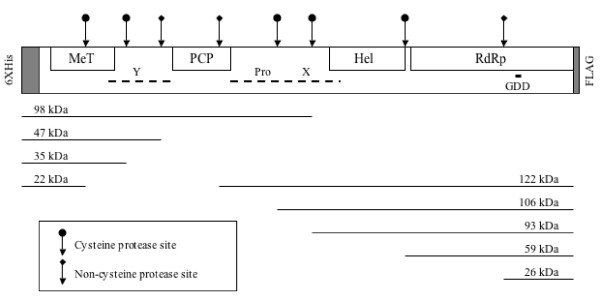
**Schematic illustration of the ORF1 polyprotein**. The illustration shows various predicted domains, the N- and C-terminal fragments detected with anti-His and anti-FLAG antibodies, respectively, and the protease cleavage sites.

The ORF1 polyprotein contains a putative PCP domain. To further validate processing of the ORF1 polyprotein and to assess the role of cysteine protease in this, we used the cell permeable cysteine protease inhibitor E-64d. Following infection of insect cells with vORF1, the cells were treated with E-64d and the cell lysates analyzed by western blotting with anti-His or anti-FLAG antibodies (Fig. 3A and 3B). At 48 hr and 60 hr post-treatment E-64d was found to inhibit ORF1 polyprotein processing as evident from accumulation of the ~192 kDa fragment (Fig. 3A and 3B, lanes 2 and 4). Western blotting with anti-His antibody revealed that addition of E-64d resulted in loss of the processed 98, 35 and 22 kDa fragments, while there was accumulation of the 47 kDa fragment at both time points (Fig. 3A, lanes 2 and 4). Under the same conditions and at similar times all processed fragments were observed in untreated cells (Fig. 3A lanes 1 and 3 respectively), while none of the fragments were seen in cells infected with the wt virus (Fig. 3A lanes 5 and 6). Equal amounts of proteins were loaded in E-64d treated, untreated or wt virus infected cells, as seen on Coomassie Blue stained gels (data not shown). The E-64d effect studied using anti-FLAG antibodies also showed accumulation of the ~192 kDa polyprotein in inhibitor-treated cells (Fig. 3B). Further, compared to untreated cells, it showed disappearance of the 106, 93 and 59 kDa fragments, with accumulation of the 122 and 26 kDa fragments (Fig. 3B, lanes 1–4). In addition, at the 60 hr time point, partially processed intermediates of ~130–140 kDa were also observed in the presence of E-64d (Fig. 3B, lane 4). As earlier, no background was observed in wt infected cells (Fig. 3B, lanes 5 and 6). Based on these results, various cysteine and non-cysteine protease sites were mapped on the ORF1 polyprotein (Fig. 4).

### Purification of protein fragments and MALDI-TOF analysis

**Figure 5:**
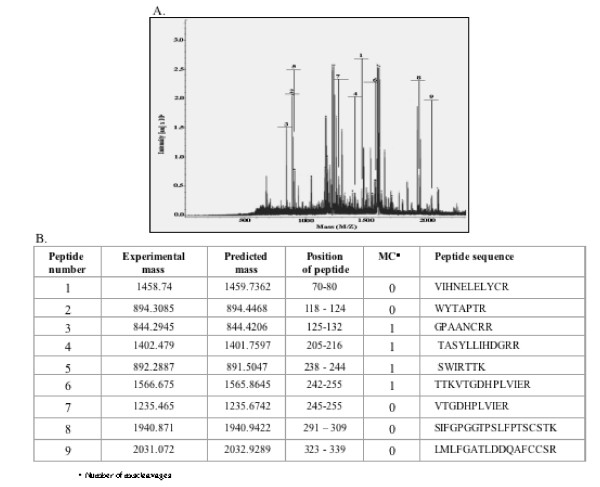
**MALDI-TOF analysis of the 35 kDa N-terminal fragment**. (A) Mass spectrum showing fragments 1–9. (B) Table shows the experimentally observed mass, predicted mass and sequences of peptides 1–9.

Protein fragments containing the His-tag were partially purified by Ni-NTA affinity chromatography. After establishing their identity using anti-His antibodies, the 35-kDa fragment was eluted from the gel and subjected to mass spectrometric analysis. Nine tryptic peptides were selected from the mass spectrum (Fig. 5A) and compared for their experimentally obtained and predicted masses (Fig. 5B). These predicted sequences matched the N- terminal region of the ORF1 polyprotein spanning amino acids 70 – 339, including the predicted MeT domain. As shown earlier (Fig. 2C), the 35-kDa fragment also stained with antibodies generated to the ORF1 MeT region spanning nucleotides 159 to 862 [16]. This antibody showed a staining pattern similar to that observed with anti-His antibodies (Fig. 2A).

## Discussion

In all plus-strand animal RNA viruses, individual proteins are processed from the nonstructural polyprotein through specific and limited proteolysis. Based on sequence homology, proposed domains and replication mechanism, HEV is closely related to alpha viruses with the Rubella virus being its closest homologue [12]. Previous studies relating to the HEV ORF1 polyprotein processing have shown that it is not processed in mammalian cells [15,16]. Despite the absence of processing, baculovirus mediated expression of a 110 kDa ORF1 protein has been shown to contain a methyltransferase activity [13]. Many mammalian proteins have been expressed in their native and active forms using recombinant baculoviruses [18]. Further, the baculovirus system has also been utilized to study the expression and processing of the polyproteins of other viruses, including the rubella viruses [19-23]. This system also offers post-translational modifications that are similar to those in mammalian cells, yet is capable of expressing much higher quantities of the recombinant protein [18]. Because of this increased signal to noise ratio, we used baculovirus-mediated expression to study HEV-ORF1 processing.

Unlike earlier reports, processing of the HEV nonstructural ORF1 polyprotein into smaller fragments was detected using antibodies to the engineered N- and C- terminal tags. A pattern of processing similar to that observed with anti-His antibodies was also observed with antibodies directed against the MeT domain. This was expected since MeT is the N-terminal domain of ORF1, and is closest to the His-tag in this construct. To further check the authenticity of processing, we performed a kinetic study of the protein expression following recombinant baculovirus infection. The ~192 kDa tagged polyprotein and at least two smaller fragments of 98 and 47 kDa appeared faintly at 24 hpi. This indicated rapid, possibly cotranslational processing since the polyhedrin promoter, under which ORF1 is placed, gets activated at around 24 hpi. The polyprotein synthesis and appearance of the processed products increased at 48 hpi and subsequent times in agreement with the characteristics of this expression system. At later times, smaller N-terminal fragments of 35 and 22 kDa were also found. This represents a precursor-product relationship, indicative of polyprotein processing. Similar observations were made when processing was monitored from the C-terminal end of the polyprotein.

Since the ORF1 polyprotein has a predicted cysteine protease domain and cis-acting proteases are found within the nonstructural polyproteins of all other positive-strand RNA viruses [23-28], it is likely that the cysteine protease within the ORF1 polyprotein is responsible for its processing. A cell-permeable cysteine protease inhibitor, E-64d, was also able to effectively block processing of the ORF1 polyprotein. Together with our kinetic data of rapid, possibly cotranslational processing of the ORF1 polyprotein, this is suggestive of a cis-acting cysteine protease within the HEV nonstructural polyprotein.

During a time course of E-64d inhibition of processing, the ~192 kDa and 47 kDa fragments observed with anti-His antibodies were found to accumulate. This suggested that cysteine protease sites occurred at 22, 35, and 98 kDa, while a non-cysteine protease site occurred at 47 kDa from the N-terminus of the tagged ORF1 polyprotein. When probed from the C-terminus with anti-FLAG antibodies, the E-64d treated cells exhibited strong accumulation of the ~192 kDa polyprotein, as well as fragments of 122 and 26 kDa. This meant that non-cysteine protease sites existed at these distances from the C-terminus of the tagged ORF1 polyprotein, while cysteine protease sites were present around 106, 93 and 59 kDa from the C-terminal end. The ~22 kDa N-terminal fragment disrupts the MeT coding region. From the present analysis, it is not clear whether this is due to nonspecific activity of the HEV protease or due to a host cell cysteine protease. Similarly, an ~26 kDa C-terminal fragment that disrupts the RdRp region is the likely product of a non-cysteine host protease. Though our results do not unequivocally prove the cysteine protease activity to have a viral origin, we clearly demonstrate ORF1 polyprotein processing. As is the case with other positive-strand RNA viruses [25-28], these results suggest a role for viral and host cell proteases in processing of the HEV ORF1 nonstructural polyprotein.

In order to further validate the processing, a 35 kDa fragment was analyzed by tryptic digestion and mass spectrometry. The results showed high confidence match with the MeT domain of ORF1 and this was confirmed by western blotting with anti-HEV MeT region antibodies.

Some earlier studies [15,16] have failed to detect ORF1 polyprotein processing. This has led Ropp *et al *[15] to speculate that the proposed cysteine protease within the HEV nonstructural polyprotein is non-functional and that HEV is different from all other positive-strand RNA viruses with respect to the processing of its nonstructural polyprotein. This has important implications for the classification of HEV within the positive-strand RNA virus group. Three lines of evidence argue against this possibility. First, using an infectious molecular clone of HEV, Panda et al [17] were able to detect proteins smaller than the 185 kDa ORF1 polyprotein with antisera prepared against recombinant methyltransferase, helicase and RdRp domains expressed in bacteria. Secondly, a 37 kDa protein identified with anti-HEV RdRp antibodies was observed in cells transfected with the HEV ORF1-EGFP replicon [29]. We present the third line of evidence in this study by demonstrating that the ORF1 polyprotein is capable of being processed and that a cysteine protease is partly responsible for this. We do understand that the baculovirus-mediated expression system employed in this study is not the natural expression system for HEV. It was used here because of our apprehension that earlier failures to observe ORF1 processing were either due to improper folding of the polyprotein expressed in prokaryotic systems, or due to low levels of expression in transfected mammalian cells. The baculovirus system offered the advantage of high expression levels and close to native post-translational modifications and protein conformation.

A comparison of all the studies on ORF1 polyprotein processing [15-17,29], including this one, also suggests the interesting possibility that polyprotein processing in the context of an infectious virus cycle [17] may require far less protein than when ORF1 is expressed on its own [15,16]. This may be due to subcellular compartmentation leading to high local concentrations of the protein precursor or due to assistance from other viral and/or cellular proteins, or some combination of these mechanisms.

When expressed using a baculovirus system, our results presented here show that even when expressed individually, the HEV ORF1 polyprotein undergoes processing. This processing is primarily mediated by a cysteine protease. Additional data is needed to conclusively establish the viral origin of this protease. To further establish this, there would be a need to over-express the ORF1 polyprotein in a mammalian cell system and to use more sensitive detection methods.

## Conclusion

While the HEV nonstructural ORF1 polyprotein carries at least four putative functional domains, its processing has so far not been demonstrated. We reasoned this may be due to improper folding or low expression levels of the polyprotein in subgenomic expression systems attempted so far. We show here expression of the ORF1 polyprotein using a baculovirus system and demonstrate processing using engineered tags, a domain-specific antibody and mass spectrometric identification of a processed fragment. A papain-like cysteine protease is predicted within the ORF1 polyprotein. We present evidence here for the role of a cysteine protease in ORF1 polyprotein processing; the viral origin of this protease remains to be established. These results have implications for the classification of HEV among positive-sense RNA viruses.

## Methods

### Materials

*Sf21 *and *T. ni *cells (Invitrogen) were maintained at 28°C in TNMFH (Gibco, BRL) and Excel 405 (JRH Biosciences) media, respectively. Antibodies to the hexahistidine and FLAG tags were purchased from Sigma. A rabbit serum containing polyclonal antibodies against the methyltransferase region of HEV ORF1 have been described earlier [16]. The Ni-NTA resin was obtained from Qiagen (Germany). All common molecular biology and cell culture grade reagents were from Sigma, unless specified otherwise.

### Construction of the ORF1 recombinant baculovirus

The ~5 kb ORF1 was PCR amplified using Gene Amp XL PCR kit (Perkin Elmer, Applied Biosystems) according to the suppliers guidelines. Besides other components, the reaction mix included 20 pmoles of each primer and 1 mM Mg(OAc)_2_. The amplification primers were designed based on alignments of the 5' and 3' ends of ORF1 in the HEV genomic sequence (GenBank Accession Number AF459438) [30]. The primers used for the amplification were EcoRI-ORF1-5', TACGGAATTCATGGAGGCCCATCAGTTTATCAAG and Hind III-ORF1-3', CCAAAGCTTTGATTTCACCCGACACAAGATTGA, containing the underlined restriction sites. The PCR amplified fragment was initially cloned in the TOPO-TA vector (Invitrogen). To position a FLAG tag at the 3'end of ORF1, the FLAG epitope was first reconstructed by annealing the oligonucleotides AGCTTAACTACAAGGACGACGACGATAAGTAACTCGAG and TCGACTCGAGTTACTTATCGTCGTCGTCCTTGTAGTCCATA. The annealed product was ligated with the vector pBBHis-2b (Invitrogen) at its HindIII and SalI sites. The PCR-amplified ORF1 fragment with EcoRI and HindIII ends was then cloned into this modified vector. The recombinant vector, pBB-ORF1 so generated, contained ORF1 flanked by hexahistidine and FLAG tags at its 5' and 3' ends respectively in a continuous reading frame (Fig. 1). The insert was sequenced to confirm the junction sequences and the translation frame before using this transfer vector for generating the recombinant baculovirus. The procedure used to construct the recombinant ORF1 baculovirus, vORF1 was essentially the same as suggested for the Bac-N-Blue DNA Transfection kit (Invitrogen). Essentially, 4 μg of recombinant plasmid (pBB-ORF1) was incubated with 0.5 μg of Bac-N-blue- DNA and Celfectin reagent (Invitrogen) at room temperature for 20 min for the formation of the DNA-liposome complex. This mixture was overlayed on *Sf21 *cells in 60 mm dishes in serum-free medium and was incubated for 4 hrs at 27°C. Following transfection, 1 ml of complete TNM-FH medium was added and incubated further at 27°C for 72 h. Recombinant virus was harvested by collecting the medium and subsequently used for two rounds of plaque purification followed by the recombinant virus amplification as described earlier [31]. This stock of virus called vORF1 was used for infection of *T. ni *cells to express ORF1 for studying its processing.

### Virus infection and analysis

To study ORF1 expression and processing, 1 × 10^6 ^*T. ni *cells were infected with 10 moi of vORF1 for 1 hour, following which the virus was replaced with Excel 405 medium. For a time-course, the infected cells were harvested at 24, 48, 60 and 72 hours post-infection (hpi). Cell lysates were prepared in SDS gel loading buffer, lysates equivalent to 30 μg of total proteins were separated by SDS-polyacrylamide gel electrophoresis (PAGE) and transferred onto a nitrocellulose membrane. Western blotting was performed with anti-His-AP conjugate (Sigma) that was detected using NBT and BCIP substrates (Gibco, BRL), or with anti-FLAG or anti-MeT antibodies. These blots were incubated with a secondary anti-rabbit or anti-mouse IgG HRP conjugate (Santa Cruz), respectively and developed using diaminobenzidine. To test for effect of the cysteine protease inhibitor E-64d, *T. ni *cells were infected with vORF1 for 12 hours, after which time the virus was removed. Fresh medium containing either E-64d dissolved in DMSO at a concentration of 200 μM or DMSO alone was added to the cells. Cells infected with wild type AcMNPV were treated similarly. The cells were allowed to grow for 48 and 60 hours post-treatment, then harvested and the lysates subjected to SDS-PAGE, followed by western blotting with anti-His or anti-FLAG antibodies as described above.

### Purification of His-tagged ORF1 fragments

*T. ni *cells in T75 flasks were infected with vORF1 at 10 moi and allowed to grow up to 48 hpi. The cells were then centrifuged, washed with PBS and stored at -80°C till further use. About 6 gm of vORF1-infected cells were suspended in 12 ml of a lysis buffer containing 50 mM sodium phosphate, pH 8.0 and 300 mM NaCl. The cells were lysed by sonication on ice, the lysates centrifuged at 14,000 rpm in a SA600 rotor (Sorvall) for 45 min at 4°C. The supernatant was collected and imidazole was added to a final concentration of 10 mM. The proteins present in the lysates were then bound with 0.5 ml of Ni-NTA resin (Qiagen, Germany) pre-equilibrated with lysis buffer, for one hour at 4°C. After binding, the resin-lysate mixture was poured into a column and washed with washing buffer containing 50 mM sodium phosphate, pH 8.0, 300 mM NaCl and 20 mM Imidazole. Following this wash, the bound proteins were eluted in 0.5 ml fractions with an elution buffer containing 50 mM sodium phosphate, pH 8.0, 300 mM NaCl and 250 mM imidazole. The purified proteins were separated by SDS-PAGE and confirmed by western blotting with anti-His antibody.

### Mass spectrometry and peptide fingerprinting

The Ni-NTA purified proteins were separated by SDS-PAGE and the gel was stained using the Silver Quest staining kit (Invitrogen). A 35 kDa band confirmed on western blot with anti-His antibody was excised and subjected to in-gel trypsin digestion and subjected to mass spectrometric analysis using a Bruker ultraflex MALDI-TOF-TOF instrument (Bruker Daltonics, Germany). The peptide Mass tool  was used to generate theoretical peptide profile of HEV ORF1 after cleaving with trypsin. These data were compared to experimentally obtained peptide masses. The MS analysis was carried out by TCGA, New Delhi.

## Competing interests

The author(s) declare that they have no competing interests.

## Authors' contributions

DS and SJ conceived of the study, analyzed the results and wrote the manuscript. DS carried out designing of primers, construction of recombinant virus and inhibition studies; ST carried out protein purification, western blots and analysis of the MALDI-TOF data; MC carried out cloning of HEV ORF1. All authors read and approved the final manuscript.
